# Prognostic impact of peripheral blood neutrophil to lymphocyte ratio in advanced-stage pulmonary large cell neuroendocrine carcinoma and its association with the immune-related tumour microenvironment

**DOI:** 10.1038/s41416-020-01188-7

**Published:** 2020-11-30

**Authors:** Masayuki Shirasawa, Tatsuya Yoshida, Hidehito Horinouchi, Shigehisa Kitano, Sayaka Arakawa, Yuji Matsumoto, Yuki Shinno, Yusuke Okuma, Yasushi Goto, Shintaro Kanda, Reiko Watanabe, Noboru Yamamoto, Shun-ichi Watanabe, Yuichiro Ohe, Noriko Motoi

**Affiliations:** 1 Department of Thoracic Oncology, National Cancer Center Hospital, 5-1-1 Tsukiji, Chuo-ku, Tokyo 104‐0045 Japan; 2 Department of Experimental Therapeutics, National Cancer Center Hospital, 5-1-1, Tsukiji, Chuo-ku, Tokyo 104-0045 Japan; 3 Department of Pathology and Clinical Laboratories, National Cancer Center Hospital East, 6-5-1, Kashiwanoha, Kashiwa-shi, Chiba 277-8577 Japan; 4 Department of Thoracic Surgery, National Cancer Center Hospital, 5-1-1, Tsukiji, Chuo-ku, Tokyo 104-0045 Japan; 5 Department of Diagnostic Pathology, National Cancer Center Hospital, 5-1-1, Tsukiji, Chuo-ku, Tokyo 104-0045 Japan

**Keywords:** Tumour immunology, Cancer microenvironment, Neuroendocrine cancer

## Abstract

**Background:**

The prognostic value of the neutrophil-to-lymphocyte ratio (NLR) with large cell neuroendocrine carcinoma (LCNEC) patients remains unclear. Thus, we performed a retrospective study to examine the relationship between the pretreatment NLR and clinical outcome in advanced LCNEC patients and the impact of the immune-related tumour microenvironment (TME).

**Methods:**

This retrospective study included 63 advanced LCNEC patients who had received chemotherapy. We collected clinical data and investigated the TME status (CD4, CD8, CD20 and FOXP3).

**Results:**

The overall survival of the patients with a low NLR (<5) was significantly longer than those with a high NLR (≥5) (14.9 vs. 5.2 months; *p* < 0.001). A multivariate analysis identified a high NLR as a predictor of a poor prognosis (HR, 3.43; 95% CI, 1.73–6.79; *p* < 0.001). The NLR was inversely correlated with tumoural and stromal CD8-positive tumour-infiltrating lymphocytes (tumoural: r = −0.648, *p* = 0.005, stromal: r = −0.490, *p* = 0.046).

**Conclusions:**

A high NLR was associated with a poor prognosis in advanced LCNEC patients. Our study revealed that the NLR can reflect the TME, at least in part, suggesting that the NLR plays an important role not only as a clinical outcome predictor but also as a tumour immune status indicator.

## Background

Lung cancer is the leading cause of cancer mortality worldwide. High-grade neuroendocrine tumour (HGNEC), which includes small cell lung cancer (SCLC) and large cell neuroendocrine carcinoma (LCNEC), accounts for 20% of all lung cancer cases.^1,2^ LCNEC is a relatively rare cancer, accounting for ~3% of primary lung cancer, but it is one of the most aggressive diseases and is characterised by widely disseminated metastases and a poor survival rate, which have been reported to be similar to those of SCLC.^3–6^ The American Society of Clinical Oncology practice guideline recommends that the standard chemotherapy regimen for LCNEC is platinum plus etoposide or the same treatment as other patients with non-squamous carcinoma.^7^ The median survival period of patients with advanced LCNEC treated with chemotherapy is 7–12 months.^3,5,8–10^

The majority of LCNEC cases are diagnosed using surgically resected specimens, since it is difficult to diagnose LCNEC using small biopsy specimens that are too small to provide sufficient morphological information for the histological criteria.^2^ However, some studies have demonstrated that diagnosis using small biopsy specimens could be feasible in terms of accuracy and availability in advanced LCNEC cases.^8,11,12^ Travis et al. proposed that “possible LCNEC” is the best term when LCNEC is strongly suspected and other diagnoses are unlikely based on the results of small biopsy samples.^2^ Therefore, in this study, we described biopsy specimens that were diagnosed as HGNEC as “possible LCNEC.”

The neutrophil-to-lymphocyte ratio (NLR) is a standard hematologic marker reflecting inflammation.^13–18^ It has also been reported to be a prognostic factor in NSCLC and SCLC patients.^19–24^ In addition, the presence of tumour-infiltrating lymphocytes (TILs) in the tumour microenvironment (TME) has been associated with the prognosis of NSCLC patients.^25–27^ However, whether the NLR and the TILs status affect the prognosis of LCNEC patients remains unknown.

Therefore, the first aim of this study is to investigate the prognostic value of NLR in advanced LCNEC patients. The second aim is to clarify the association between NLR, an indicator of inflammation in peripheral blood, and the status of TILs in TME, which reflects inflammation in tumour tissue.

## Methods

### Study patients

This retrospective study enrolled patients who were pathologically diagnosed as having advanced LCNEC or advanced possible LCNEC and who received systemic chemotherapy at the National Cancer Center Hospital (Tokyo, Japan) between January 2001 and December 2018. In accordance with the 2015 WHO classification, a diagnosis of LCNEC was made when surgical specimens were available. LCNEC patients who met the following 2015 WHO criteria were eligible for enrolment in this study: (1) non-small-cell carcinoma (NSCLC) with a neuroendocrine (NE) morphology (organoid nesting, palisading, trabeculae and/or rosettes), (2) presence of necrosis or mitosis (>10 mitoses per 2 mm^2^ [with an average of 75] in a surgical specimen) and (3) at least one positive NE marker (chromogranin-A, synaptophysin, CD56/NCAM).^2^

When only small biopsy samples were available, a diagnosis of “possible LCNEC” was made if the following criteria were met: (1) poorly differentiated NSCLC with an NE morphology; (2) presence of high-grade features such as mitosis, necrosis or high Ki67 LI and (3) presence of NE differentiation.^28^ We excluded the possibility of carcinoid tumour and small cell carcinoma, although the possibility of combined small cell carcinoma and LCNEC cannot be excluded because of the limited sampling. In this study, all the specimens were diagnosed by at least two expert lung pathologists at the time of the original diagnosis. Also, two of the co-authors, the expert lung pathologists (RW, NM), reviewed and confirmed the diagnoses of LCNEC or possible LCNEC based on both morphological and immunohistochemical features (Supplemental Fig. 1).

### Collection of clinical data

The following data were collected: stage classified according to the 8th TNM classification,^29^ age at which chemotherapy was initiated, sex, smoking status, the Eastern Cooperative Oncology Group performance status (PS), and laboratory data (including levels of NLR, albumin, lactate dehydrogenase [LDH], C-reactive protein [CRP], neuron-specific enolase [NSE] and pro-gastrin-releasing peptide [Pro-GRP]) obtained before the initial chemotherapy. NLR was defined as the total neutrophil count divided by the total lymphocyte count. The upper limit of normal levels (UNL) for LDH was defined as ≥229 U/L, and the UNL for CRP was defined as ≥0.1 mg/dL. We determined the NLR cut-off value to be 5, based on a previous study examining SCLC.^21^ Progression-free survival (PFS) was defined as the interval from the beginning of chemotherapy to the date of disease progression or death, whichever occurred first. Patients without any of these events were censored at the final follow-up as being without documented progression. Overall survival (OS) was defined as the time from the beginning of chemotherapy to the date of death from any cause. Patients without any of these events were censored at the final follow-up as being without documented progression. The data cut-off date was August 10, 2019. Among the LCNEC patients, the time of recurrence was set as the date of diagnosis, and we used the pretreatment data that was collected during the first round of chemotherapy after recurrence.

### Processing of tissue specimens

We created tissue microarrays (TMA) using suitable surgically resected specimens. The TMA was built with TMA Master^®^ (Beacher Biotech) using 2-mm cores from two representative areas obtained from each case. The histology of each core from each case was evaluated by a board-certified lung pathologist (NM).

### Immunohistochemical staining

Immunohistochemical staining (IHC) was performed according to the manufacturer’s instruction using an autostainer (Dako autostainer Link 48 and Omnis staining platform; Dako, Glostrup, Denmark) and the following monoclonal antibodies: PD-L1 (PD-L1 IHC [22C3] pharmDx; Agilent), CD4 (clone 4B12; Novocastra), CD8 (clone 4B11; Novocastra), CD20 (L26; Thermo) and FOXP-3 (236 A/E7; Abcam).

### Evaluation of tumour-infiltrating immune cells

To evaluate the TILs status in the TME, the stained slides were scanned at ×40 magnification and digital images were created using the NanoZoomer Digital Pathology (NDP) system (NanoZoomer 2.0-HT Whole Slide Imager; Hamamatsu Photonics, Hamamatsu, Japan). Using the resulting digital images, hot spots of immune cell infiltration were evaluated by two observers (MS and NM) who were blinded to all of the clinical data. The immune cell number was counted, and the areas of tumour and stroma were measured using NDP.view2 (version 2.6; Hamamatsu Photonics). For hot spots, the degree of tumour-infiltrating lymphocytes (CD4, CD8, CD20 and FOXP3) was calculated as the number of positive cells divided by the examined area (per 1 square millimetre). The TILs were divided into tumoural and stromal ones based on their location. Tumoural TILs were defined as lymphocytes that had infiltrated the tumour cell nest. Stromal TILs were defined as lymphocytes that had infiltrated the stromal tissue, including both inter-tumoural and the surrounding connective tissue beside the tumour (Supplemental Fig. 2).

### Statistical analyses

We analysed the categorical data and differences in the clinical and laboratory data between the two groups using the χ^2^ test and the *t*-test, and the correlation between two continuous variables using the Spearman test. All survival analyses were performed using the Kaplan–Meier method. In addition, the differences in survival times between the groups based on prognostic factors were compared using the log-rank test. Spearman’s rank test was used to analyse statistically the correlation between the NLR and the numbers of immune cells showing positive reactions with antibodies (CD4, CD8, CD20 and FOXP3). We used a Cox proportional hazards model for univariate and multivariate analyses to identify the prognostic factors. All the analyses were performed using the SPSS software program, version 19 (SPSS Inc. Chicago, IL, USA). This study was approved by the Ethics Committee of the National Cancer Center Hospital (2015-289, 2018-264, 2019-123).

## Results

### Patient characteristics

There were 63 patients with the histologically proven disease during the study period: 27 surgically resected LCNEC patients, and 36 possible LCNEC patients whose diagnoses were made based on biopsied specimens. The median age was 62 years (range, 28–79 years); 55 patients (87.3%) were male, and only four patients (6.3%) were non-smokers. According to the 8th TNM staging system, 6 (9.5%), 30 (47.6%) and 27 (42.9%) patients were categorised into stages IIIB, IV and postoperative recurrence, respectively. Patients with stage IIIB or IV disease were diagnosed as possible LCNEC, and patients with postoperative recurrence were diagnosed as LCNEC because of the availability of a surgical sample. In this study, 5 of the 27 patients who underwent surgery received postoperative adjuvant chemotherapy. The median time to relapse of the LCNEC patients who relapsed after surgery was 9.2 (4.3–14.0) months. Forty-seven patients (74.6%) had low NLR values (<5) before the initial chemotherapy. The clinical characteristics of the patients according to the NLR value (<5 or ≥5) are shown in Table 1. The levels of albumin and CRP differed significantly between the high NLR group and the low NLR group (albumin: *p* = 0.01, CRP: *p* = 0.02).

**Table 1:** Patient characteristics according to NLR (<5 or ≥5) in this study (*n* = 63).

	All patients	NLR < 5 *n* = 47	NLR ≥ 5 *n* = 16	*p*
Age, median (range), in years	62 (28–79)	63 (28–79)	62 (48–79)	
Age, *n* (%)				
<75 years	56 (88.9)	42 (89.4)	14 (87.5)	
≥75 years	7 (11.1)	5 (10.6)	2 (12.5)	1.00*
Gender, *n* (%)				
Male	55 (87.3)	41 (87.2)	14 (87.5)	
Female	8 (12.7)	6 (12.8)	2 (12.5)	1.00*
Smoking status, *n* (%)				
Never	4 (6.3)	3 (6.4)	1 (6.2)	
Former/current	59 (93.7)	44 (93.6)	15 (93.8)	1.00*
ECOG PS, *n* (%)				
0–1	60 (95.2)	46 (97.8)	14 (87.5)	
2/3–4	3 (4.8)/0 (0)	1 (2.2)/0 (0.0)	2 (12.5)/0 (0.0)	0.16*
Stage, *n* (%)				
IIIB	6 (9.5)	5 (10.6)	1 (6.2)	
IV	30 (47.6)	21 (44.7)	9 (56.3)	
Recurrence	27 (42.9)	21 (44.7)	6 (37.5)	0.77*
Brain metastasis, *n* (%)				
Yes	14 (22.2)	8 (17.1)	6 (37.5)	
No	49 (77.8)	39 (82.9)	10 (62.5)	0.16*
Blood tests, average ± SD				
Alb, g/dL	3.9 ± 0.5	4.0 ± 0.4	3.5 ± 0.6	0.01**
LDH, IU/L	473 ± 707	389 ± 400	719 ± 1219	0.31**
CRP, mg/dL	1.7 ± 3.4	0.8 ± 1.5	4.4 ± 5.6	0.02**
NSE, ng/mL	47.3 ± 67.0	46.4 ± 74.1	46.9 ± 40.7	0.86**
Pro-GRP, pg/mL	704 ± 3757	893 ± 4350	158 ± 444	0.50**

*NLR* neutrophil-to-lymphocyte ratio, *ECOG* Eastern Cooperative Oncology Group, *Alb* albumen, *LDH* lactate dehydrogenase, *CRP* C-reactive protein, *NSE* neuron-specific enolase, *pro-GRP* pro-gastrin-releasing peptide

*p values were analysed using the χ² test.

**p values were analysed using the *t*-test.

### Clinical outcomes in all the patients (LCNEC and possible LCNEC patients)

**Fig. 1:**
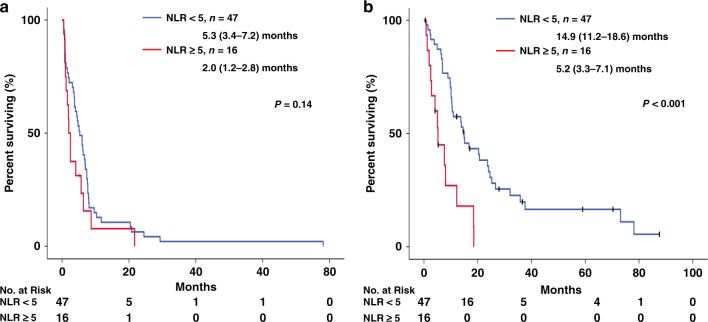
Kaplan–Meier analysis of PFS and OS according to NLR value (*n* = 67). **a** PFS and **b** OS in all patients with a low NLR (blue) vs. patients with a high NLR (red). *P* values were determined using the log-rank test; the number of individuals in each group and the median survival time (95% CI) are indicated.

Thirty-seven patients (58.7%) received platinum and irinotecan (CPT) and 16 patients (25.4%) received platinum and etoposide (ETP) as their first-line chemotherapy regimen. The details of the first-line chemotherapy according to the NLR value are presented in Table 2. Regarding the response rate after first-line chemotherapy, the difference between patients with a low NLR (<5) and those with a high NLR (≥5) was not significant (48.9% vs. 28.6%, *p* = 0.23). The PFS of first-line chemotherapy was 4.6 months (95% confidence interval [CI], 2.8–6.4 months). The patients with a low NLR tended to have a better PFS than those with a high NLR during first-line chemotherapy (5.3 months [95% CI, 3.4–7.2] vs. 2.0 months [95% CI, 1.2–2.8]; *p* = 0.14; Fig. 1a). The median OS of all the patients (*n* = 63) was 12.2 months (95% CI, 7.8–16.6 months). Patients with a low NLR had a significantly better OS than those with a high NLR (14.9 months [95% CI, 11.2–18.6] vs. 5.2 months [95% CI, 3.3–7.1]; *p* < 0.001; Fig. 1b).

**Table 2:** Details of first-line chemotherapy in patients with LCNEC of NLR < 5 or NLR ≥ 5 (*n* = 67).

	NLR < 5 *n* = 47	NLR ≥ 5 *n* = 16	p*
Regimen of initial chemotherapy, *n* (%)			
Platinum + CPT	29 (61.7)	8 (50)	
Platinum + ETP	10 (21.3)	6 (37.5)	
Platinum + PTX	3 (6.4)	0 (0.0)	
AMR	3 (6.4)	2 (12.5)	
Other	2 (4.2)	0 (0.0)	
Cycles of chemotherapy, median (range)	2.0 (1–8)	1.0 (1–5)	
Response to initial chemotherapy, *n* (%)			
Partial response	23 (48.9)	4 (28.6)	0.23
Stable disease	11 (23.4)	5 (35.7)	
Progressive disease	13 (27.7)	5 (35.7)	
Not evaluated	0	2	

*NLR* neutrophil-to-lymphocyte ratio, *CPT* irinotecan, *ETP* etoposide, *PTX* paclitaxel, *AMR* amrubicin.

*p values were analysed using the χ² test.

Next, we analysed the outcomes of the LCNEC and possible LCNEC patients separately. Among the LCNEC patients, the patients with a low NLR (<5) tended to have a better outcome than the patients with a high NLR (≥5) (15.1 [95% CI, 7.9–22.3] vs. 2.5 [95% CI, 0.0–19.0] months; *p* = 0.09; Supplemental Fig. 3B). Among the possible LCNEC patients, the OS of the patients with a low NLR (<5) was significantly better than that of the patients with a high NLR (≥5) (13.7 [95% CI, 6.3–21.1] vs. 5.2 [95% CI, 3.8–6.6] months; *p* < 0.001; Supplemental Fig. 3D).

### Survival analyses with consideration of the clinical characteristics

In a univariate analysis of patients with LCNEC and possible LCNEC, a high NLR (≥5) was a predictor of an unfavourable prognosis (hazard ratio [HR], 3.43; 95% CI, 1.73–6.79; *p* < 0.001; Table 3). Multivariate analysis confirmed that a high NLR (≥5) was a predictor of an unfavourable prognosis regardless of the type of LCNEC (possible LCNEC vs LCNEC) (HR, 3.53; 95% CI, 1.74–7.17; *p* < 0.001; Table 3).

**Table 3:** Survival analysis in patients with LCNEC (*n* = 67).

	Univariate			Multivariate		
	HR	95% CI	*p*	HR	95% CI	*p*
Age ≥75 vs. <75	0.53	0.19–1.48	0.22	0.65	0.22–1.95	0.65
Sex Female vs. male	1.30	0.58–2.91	0.52	1.46	0.57–3.73	0.43
Smoking status Former/current vs. never	0.82	0.29–2.31	0.71	0.94	0.28–3.08	0.91
ECOG performance status 2–4 vs. 0–1	1.75	0.54–5.68	0.35	1.62	0.48–5.47	0.44
Stage Stage IIIB, IV vs. recurrence (possible LCNEC vs. LCNEC)	0.87	0.49–1.54	0.63	0.90	0.45–1.77	0.75
Brain metastasis Yes vs. no	0.96	0.48–1.92	0.90	0.83	0.39–1.80	0.64
NLR ≥5.0 vs. <5.0	3.43	1.73–6.79	<0.001	3.53	1.74–7.17	<0.001

*ECOG* Eastern Cooperative Oncology Group, *LDH* lactate dehydrogenase, *LDH* lactate dehydrogenase, *NLR* neutrophil-to-lymphocyte ratio.

### Association between NLR and clinicopathologic parameters

**Fig. 2:**
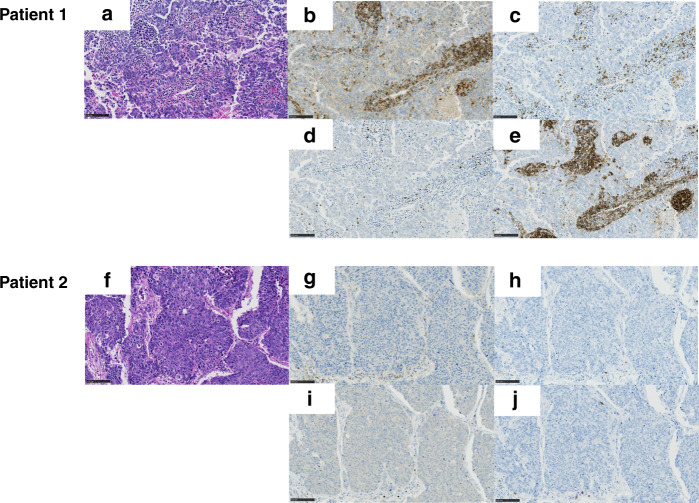
Overview of pathology slides in two patients. Representative images of pathology slides showed a high-density positive TILs tumour (upper) and a low-density positive TILs tumour (lower) in patients with LCNEC. HE-positive (**a** and **f**), CD4-positive (**b** and **g**), CD8-positive (**c** and **h**), FOXP3-positive (**d** and **i**) and CD20-positive (**e** and **j**) TILs in the tumour nest and stroma area are visible (Bar = 50 μm).

**Fig. 3:**
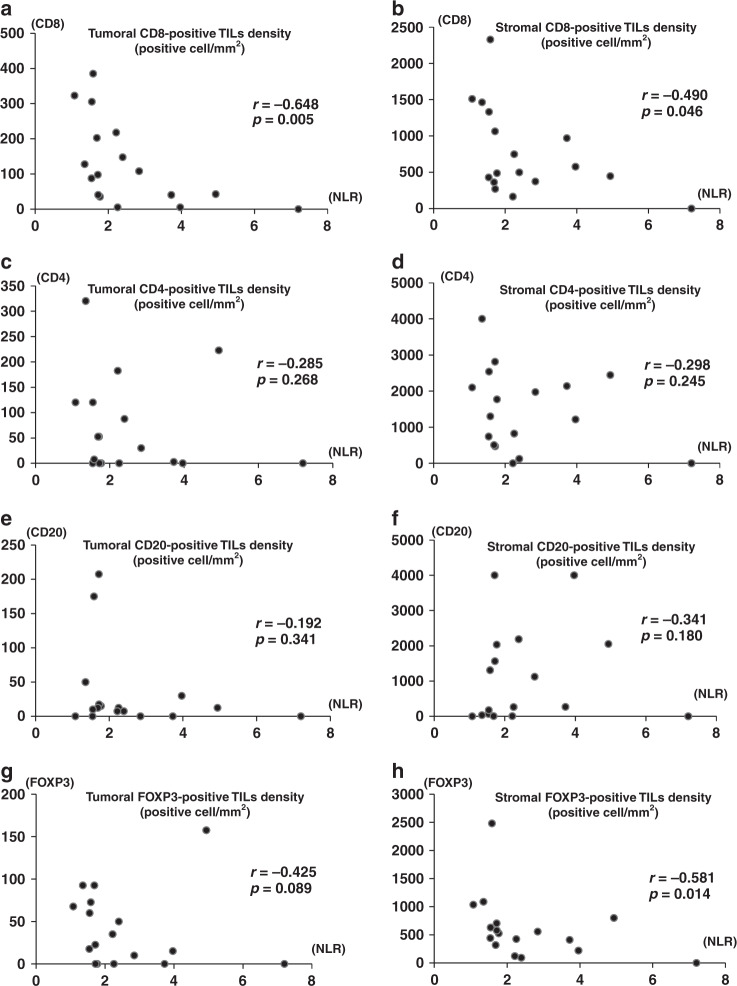
Relation between the NLR in preoperative blood tests and the TILs status in the TME. Relation between the NLR value in preoperative blood tests and the density of tumoural and stromal CD8-positive (**a** and **b**), CD4-positive (**c** and **d**), CD20-positive (**e** and **f**) and FOXP3-positive (**g** and **h**) cells in the TME.

Eighteen out of 27 surgically resected specimens were suitable for the creation of a TMA. To evaluate whether the NLR values were correlated with the TILs status in TME, we performed IHC for CD4, CD8, CD20 and FOXP3 (Fig. 2). The correlations between the NLR values at baseline and the TILs statuses in patients with LCNEC are described in Fig. 3. A high NLR in preoperative blood samples was significantly and negatively correlated with tumoural (r = −0.648, *p* = 0.005) and stromal (r = −0.490, *p* = 0.046) CD8-positive TILs and the stromal (r = −0.581, *p* = 0.014) FOXP3-positive TILs density, compared with a low NLR.

## Discussion

We retrospectively surveyed 63 patients with advanced LCNEC who had received systemic chemotherapy. Our study revealed that LCNEC patients with a high NLR at baseline had a significantly poorer outcome than those with a low NLR.

The NLR is reportedly associated with inflammation.^13–18,30,31^ In this study, the levels of albumin and CRP, which are related to inflammation, differed significantly between the high NLR group and the low NLR group (albumin: *p* = 0.01, CRP: *p* = 0.02).^32,33^ Inflammation increases the number of neutrophils and affects tumour growth and progression. In general, neutrophils suppress lymphocytes.^34,35^ In an in vitro platform, neutrophils in a TME induced apoptosis in lymphocytes, such as CD8-positive T cells, in a contact-dependent manner.^36^ In contrast, lymphocytes are essential immune cells in both humoral and cellular antitumour immune responses, and low lymphocyte counts are associated with a general suppression of the immune system in cancer patients.^37–40^ Therefore, a high NLR has been considered to be a predictor of a poor prognosis for various cancers.^19–24,41–43^ Indeed, a meta-analysis of NSCLC patients demonstrated that a high NLR predicted a more inferior OS and PFS.^24^ In extensive disease SCLC, which is a neuroendocrine carcinoma in the lung, a high NLR was also a predictor of a poor prognosis.^21^ No data is available regarding the relationship between the pretreatment NLR and the clinical outcome in patients with advanced LCNEC.

On the other hand, how NLR values are correlated with the immune-related TME remains unclear. We found that the NLR value in peripheral blood was correlated with the status of CD8-positive TILs in the TME, but not with the expression of PD-L1 in tumour cells and immune cells expressing other markers including CD4, and CD20. CD8-positive T lymphocytes take part in cancer immunity through their ability to kill tumour cells via major histocompatibility complex locus class I (MHC-I)/β-2-microglobulin complexes. The status of CD8-positive TILs in the TME is associated with the prognosis of various cancers.^25–27^ Therefore, the inverse correlation observed between the density of CD8-positive TILs and the NLR value is consistent with the finding that the NLR value was a prognostic factor in LCNEC patients.

In our study, stromal FOXP3-positive cells were also negatively correlated with the NLR value. The FOXP3 transcription factor generally acts on regulatory T (Treg) cells, preventing an effective immune response against the tumour.^44,45^ The prognostic significance of FOXP3 expression remains controversial. Previous studies have shown that FOXP3 expression is associated with a poor prognosis in patients with various cancers.^46–52^ On the other hand, a meta-analysis on gastric cancer described that tumoural Foxp3-positive TILs were associated with poor survival, whereas extratumoural Foxp3-positive T-cell invasion was associated with better survival, suggesting that FOXP3 T cells have opposite functions in the intra- and extratumoural environments.^53^ In fact, in LCNEC patients, stromal FOXP3 expression on TILs has been reported to be a favourable prognostic factor.^53^

Additionally, tumour-infiltrating FOXP3-positive T cells can be classified into two types according to the degree of the FOXP3 expression level in colorectal cancer patients.^54^ These functionally distinct subpopulations of tumour-infiltrating Foxp3-positive T cells contribute in opposing ways to determining prognosis. Further investigation of the types of T cells with FOXP3 expression and the FOXP3 expression levels on TILs might be needed.

Based on the results of our study, the pretreatment NLR value appears to be a useful measurement that reflects the prognosis of patients with advanced LCNEC. Additionally, PD-1 blockade has recently become a standard therapy against multiple cancers. In NSCLC patients, PD-L1 expression on tumour cells is a potential predictor of PD-1 blockade, but PD-L1 expression in LCNEC patients was lower than in patients with other types of NSCLC^55–57^ and reliable predictive markers of anti-PD-(L)1 therapy are lacking in LCNEC patients. In this study, we analysed the correlation between the NLR of the preoperative blood test and the NLR of the recurrence blood test in an exploratory manner. The NLR of the preoperative blood test, which was correlated with the CD8-positive TILs status, was positively correlated with the NLR of the recurrence blood test (Spearman test: correlation coefficient, 0.740; *p* = 0.001; Supplemental Fig. 4). Therefore, the NLR of the recurrence blood test could be a predictor of the efficacy of PD-1 blockade in LCNEC patients.

This study had some limitations. First, this was a retrospective study with a small sample size conducted at a single institution. There was a selection bias of chemotherapy between LCNEC patients and possible LCNEC patients. In this study, most patients (84.1%) were treated with CDDP + CPT or CDDP + ETP. However, five LCNEC patients who received postoperative adjuvant chemotherapy after surgery were treated with AMR as an initial chemotherapy regimen. Second, this study included patients with advanced possible LCNEC who had been diagnosed using small biopsy samples. In general, the diagnosis of possible LCNEC using small biopsy specimens is difficult and based on the combination of NSCLC with a NE morphology and positivity of NE markers. As for NE markers, 11 cases of possible LCNEC (30.5%) were positive for all three NE markers (synaptophysin, chromogranin-A, and CD56), 17 cases (47.2%) were positive for two markers and 8 cases (22.5%) were positive for one marker. In past reports, positive staining for greater than or equal to two of the three neuroendocrine IHC markers was capable of distinguishing between LCNEC and NSCLC with a sensitivity and a specificity of 80% and 99%, respectively.^58^ Baine, et al., recently proposed a scoring combining three NE markers and morphology for the diagnosis of LCNEC.^28^ Utilising a cut-off score of 4 or higher yielded a 100% sensitivity and a 99% specificity for the diagnosis of LCNEC, with an excellent agreement among four pathologists (98%). Among the 36 possible LCNEC cases in our study, all 36 cases had a score of 4 or higher. In addition, we evaluated the mitosis and Ki67 expression on tumour cells, if specimens were available. The presence of nuclear mitosis was confirmed in all 36 possible LCNEC patients. The median (range) frequency of Ki67 expression in the evaluable cases was 80% (50–90%), which was higher than that in NSCLC cases.^1^ Therefore, in terms of NE markers, mitosis and Ki67 expression, our cases in this study were consistent with possible LCNEC.

Next, we analysed the clinical outcomes of LCNEC and possible LCNEC patients separately. In both the LCNEC and the possible LCNEC patients, the PFS and the OS in the patients with a low NLR tended to be longer than those with a high NLR (Supplemental Fig. 3). Additionally, no significant difference in either the PFS or the OS was seen between the LCNEC patients and the possible LCNEC patients (PFS: 6.4 months [95% CI, 1.5–11.3] vs. 4.1 months [95% CI, 2.3–5.9]; *p* = 0.25, and OS: 14.9 months [95% CI, 4.7–25.1] vs. 10.5 months [95% CI, 9.4–11.6]; *p* = 0.63). Thus, the clinical features between LCNEC and possible LCNEC were similar. However, it remains unclear whether the use of the same category for the two groups (LCNEC and possible LCNEC) is relevant. Further investigation of the differences in clinical and pathological backgrounds between possible LCNEC and LCNEC is needed, although obtaining sufficient tissue samples from possible LCNEC patients is difficult.

Third, we showed an inverse correlation between the density of CD8-positive TILs and the NLR in advanced LCNEC patients, but no data are available for other malignancies, especially NSCLC and SCLC. Further study is needed to examine the differences in the TME between LCNEC and other subsets of lung cancer.

In conclusion, a high NLR value was significantly associated with a poor outcome and the presence of tumoural and stromal CD8-positive TILs in the TME in patients with advanced LCNEC.

## Supplementary information

Supplemental Figure 1, 2, 3, 4

## Data Availability

The datasets used and/or analysed during the current study are available from the corresponding author on reasonable request.
