# Supplementary material for: Prognostic impact of peripheral blood neutrophil to lymphocyte ratio in advanced-stage pulmonary large cell neuroendocrine carcinoma and its association with the immune-related tumour microenvironment
Source: Br J Cancer. 2020 Nov 30;124(5):925–32. doi: 10.1038/s41416-020-01188-7 (PMC7921668; doi:10.1038/s41416-020-01188-7)
Supplement: Supplementary file 1 — Supplemental Figure 1, 2, 3, 4 [file 41416_2020_1188_MOESM1_ESM.pdf]

# Supplemental Figure 1.

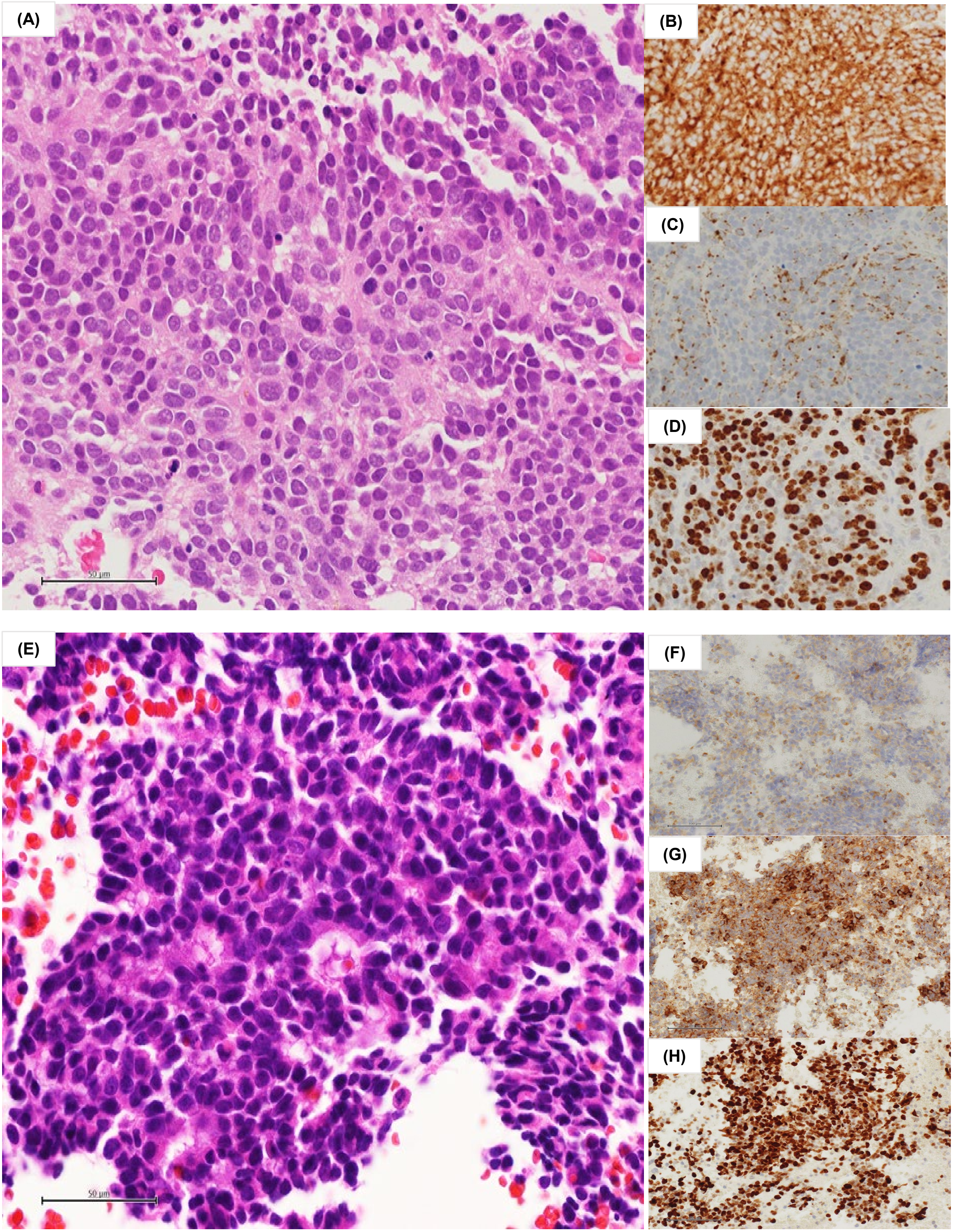

# Supplemental Figure 2.

**Patient 1, CD8**

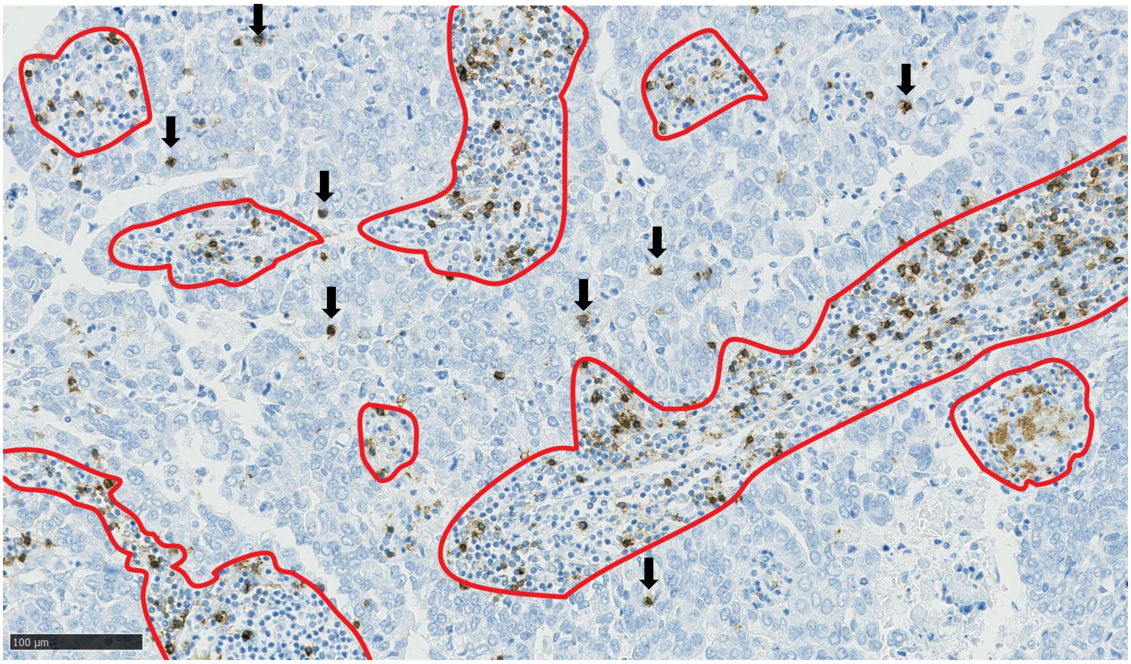

# Supplemental Figure 3.

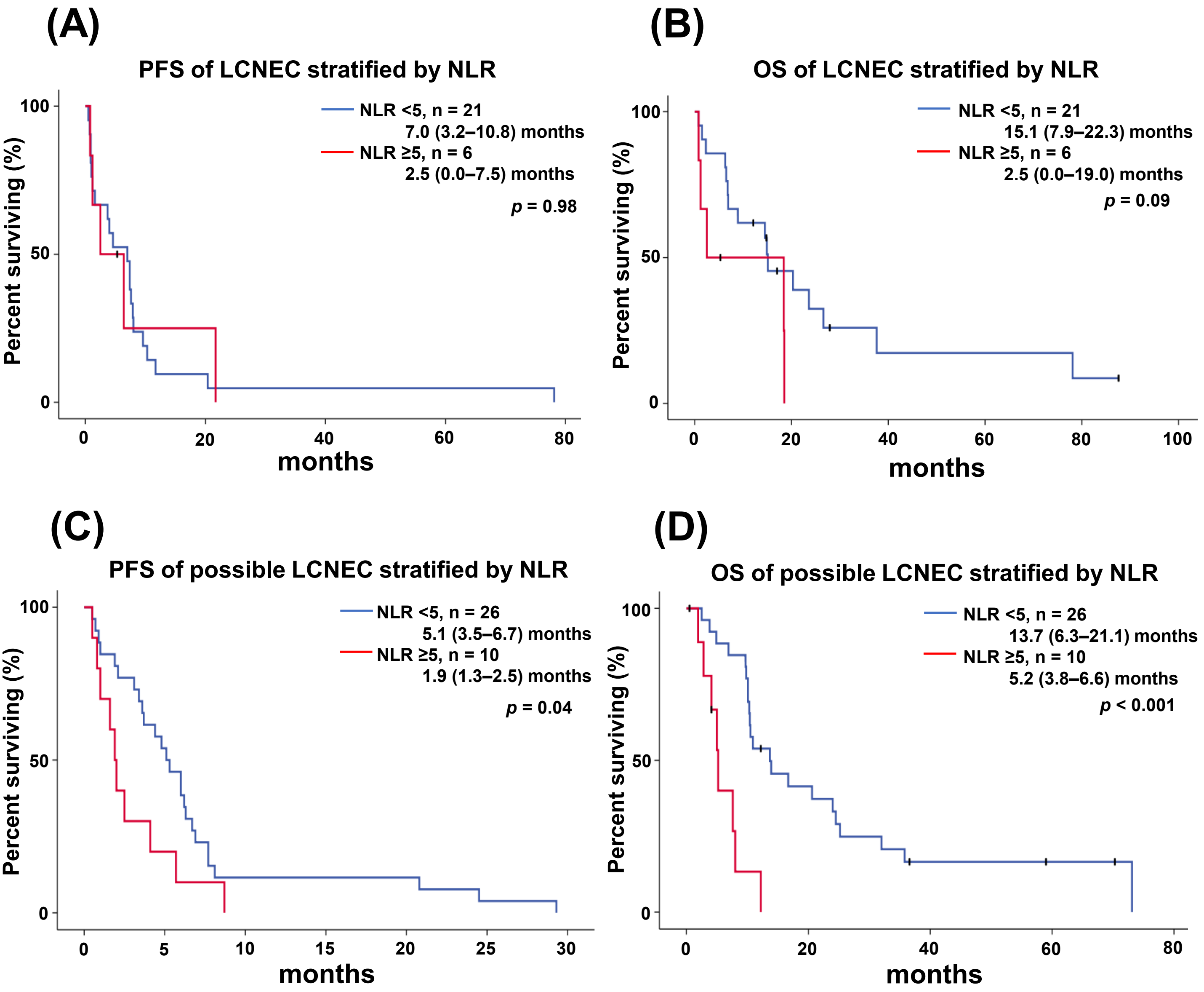

# Supplemental Figure 4.

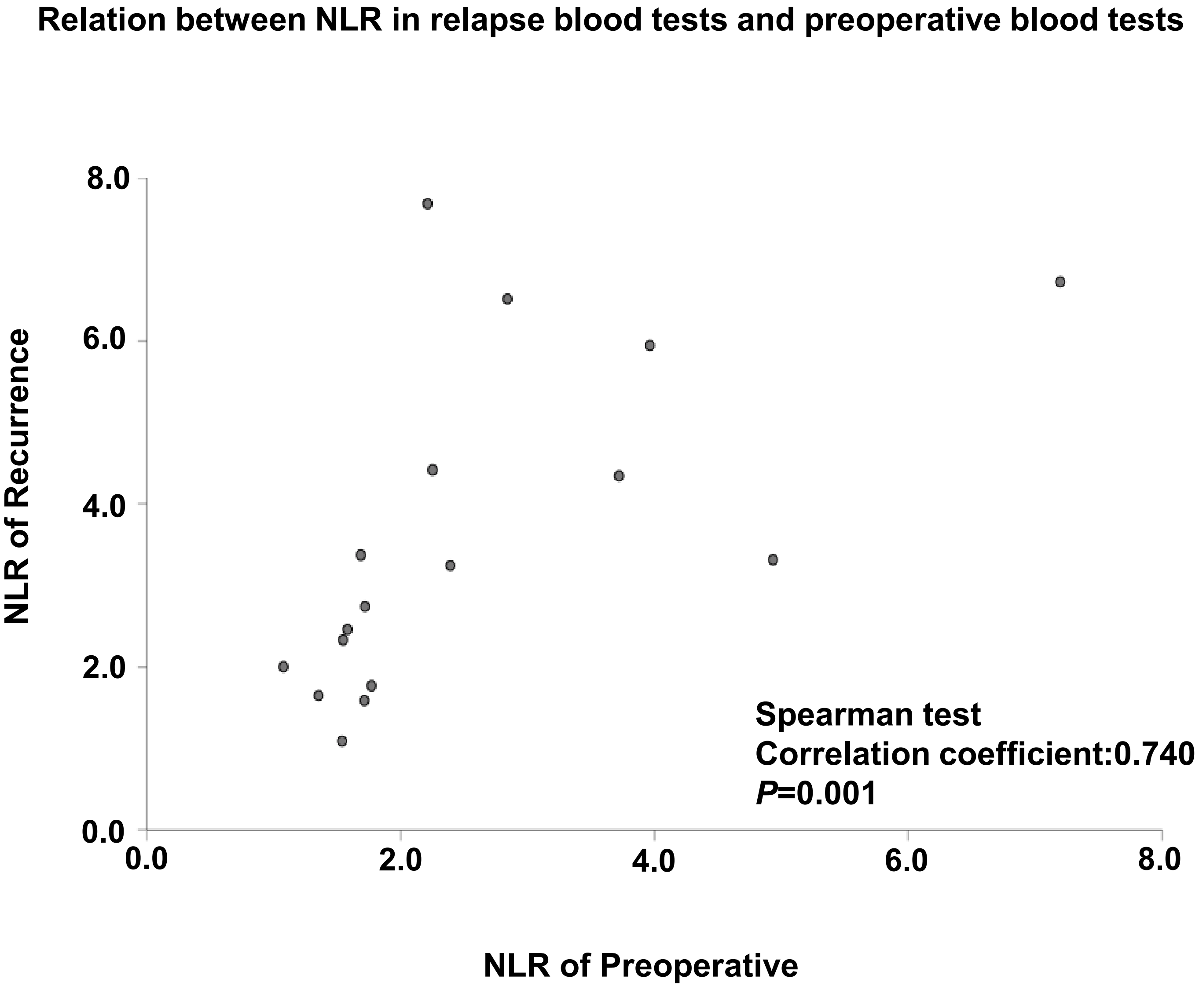

.

**Supplemental Figure Legends**

**Supplemental Figure 1.**

Representative images of pathology slides from patients with possible LCNEC. All the cases are obviously non-small cell carcinoma with distinct cytoplasm features, showing a rosette-like, organoid structure indicating neuroendocrine differentiation on HE-stained slides (A, and E). All the cases showed the immunoreactivity of two or more NE markers (synaptophysin [B, and F], chromogranin A [C, and G], and a high Ki-67 labeling index [D, and H]). One specimen (A-D) was obtained by needle biopsy, and the other (E-H) was obtained by transbronchial biopsy.

**Supplemental Figure 2.**

Representative image of a pathology slide for LCNEC. The area surrounded by the red line shows the stroma. Tumoral CD8-positive TILs are shown by the arrowheads.

**Supplemental Figure 3.**

Kaplan–Meier analysis of (A, and C) PFS and (B, and D) OS in LCNEC and possible LCNEC patients with a low NLR (blue) vs. patients with a high NLR (red). *P* values were determined using the log-rank test; the number of individuals in each group and the median survival time (95% CI) are indicated.

**Supplemental Figure 4.**

.

Relation between the NLR value in preoperative blood tests and recurrence blood test.
